# Supplementary material for: WNT inhibition creates a BRCA‐like state in Wnt‐addicted cancer
Source: EMBO Mol Med. 2021 Mar 4;13(4):e13349. doi: 10.15252/emmm.202013349 (PMC8033517; doi:10.15252/emmm.202013349)
Supplement: Supplementary file 1 — Expanded View Figures PDF [file EMMM-13-e13349-s002.pdf]

Expanded View Figures

Figure EV1. (accompanying Figs 1 and 2).

A, B Olaparib and ETC-159 synergize in multiple Wnt-addicted cancer cells. Soft agar colony formation assays were performed as in Fig 1A with the indicated cell lines treated with varying concentrations of ETC-159, olaparib, or a combination of both. Representative image of soft agar colonies of (A) MCAS and (B) CFPAC-1 cells is shown.

C Timeseries analysis clusters genes into distinct patterns based on their transcriptional response to PORCN inhibition. Reanalysis of data from (Madan *et al*, 2018), where HPAF-II cells were orthotopically injected into the tail of the pancreas. Tumors were established over a period of 28 days, and mice were treated with ETC-159 (37.5 mg/kg *bid*). RNA was isolated from the tumors at the indicated time points and analyzed by RNA-seq. The heatmap shows all genes that were differentially expressed over time (FDR < 10%) following PORCN inhibition, clustered into 64 clusters based on their pattern of transcriptional response. The clusters of genes that are robustly downregulated following Wnt inhibition are highlighted with colors and indicated as *Wnt-activated* genes.

D ETC-159 treatment of HPAF-II tumors downregulates protein levels of BRCA1. Tumor lysates from HPAF-II xenografts treated with vehicle or ETC-159 for 56 h were analyzed by SDS–PAGE and immunoblotted with the anti-BRCA1 antibody. Each lane represents an individual tumor.

E Wnt inhibition does not alter the cell cycle phases in HPAF-II cells. HPAF-II cells were treated with DMSO or ETC-159 for 48 h. After treatment, cells were stained with propidium iodide and analyzed using flow cytometry to determine the number of cells in G_1, S, or G_2/M phase of the cell cycle. Each bar represents mean ± SD of two replicates.

F Wnt inhibition reduces the expression of HR and FA pathway genes in HPAF-II cells. HPAF-II cells were treated with DMSO or ETC-159 (100 nM) for 48 h. Total RNA was isolated, and the normalized expression of DNA repair genes as measured by RNA-seq is shown. The horizontal lines represent mean of replicates.

G Wnt inhibition reduces the expression of HR and FA pathway genes in Wnt high EGI-1 cells. EGI-1 cells were cultured in low adherence plates and treated with DMSO or ETC-159 (100 nM) for 72 h. Total RNA was isolated, and the expression of *AXIN2* and DNA repair genes was measured by qRT–PCR. The horizontal lines represent mean of replicates.

H Wnt inhibition does not alter the cell cycle phases in AsPC-1 cells. AsPC-1 cells were treated with DMSO or ETC-159 for 48 h. After treatment cells were stained with propidium iodide and analyzed using flow cytometry to determine the number of cells in G_1, S, or G_2/M phase of the cell cycle. Each bar represents the mean ± SD of two replicates.

© 2021 The Authors

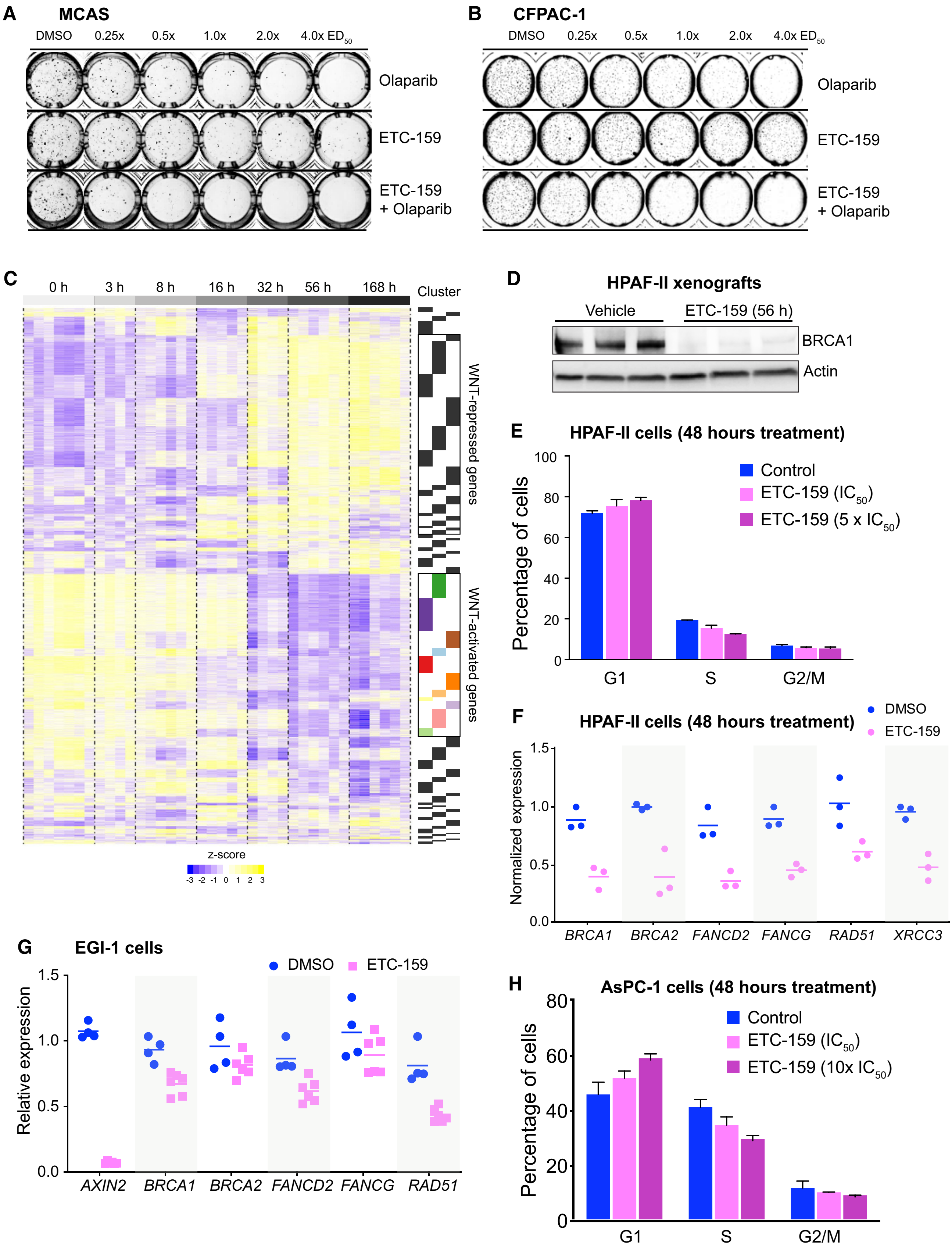
A
MCAS
DMSO 0.25x 0.5x 1.0x 2.0x 4.0x ED50
Olaparib
ETC-159
ETC-159 + Olaparib
B
CFPAC-1
DMSO 0.25x 0.5x 1.0x 2.0x 4.0x ED50
Olaparib
ETC-159
ETC-159 + Olaparib
C
0 h 3 h 8 h 16 h 32 h 56 h 168 h
Cluster
WNT-repressed genes
WNT-activated genes
z-score
-3 -2 -1 0 1 2 3
D
HPAF-II xenografts
Vehicle
ETC-159 (56 h)
BRCA1
Actin
E
HPAF-II cells (48 hours treatment)
Percentage of cells
Control
ETC-159 (IC50)
ETC-159 (5 x IC50)
G1 S G2/M
F
HPAF-II cells (48 hours treatment)
DMSO
ETC-159
Normalized expression
BRCA1 BRCA2 FANCD2 FANCG RAD51 XRCC3
G
EGI-1 cells
DMSO ETC-159
Relative expression
AXIN2 BRCA1 BRCA2 FANCD2 FANCG RAD51
H
AsPC-1 cells (48 hours treatment)
Percentage of cells
Control
ETC-159 (IC50)
ETC-159 (10x IC50)
G1 S G2/M

Figure EV1.

Figure EV2. (accompanying Fig 5).

A Temporal regulation of *FOXM1* in HPAF-II orthotopic xenografts treated with ETC-159. Each data point represents an individual tumor. The horizontal line represents the median with the lower and upper edges of the boxes representing 25th and the 75th percentile of the data, respectively, and the whiskers representing 1.5× interquartile range.

B Expression of DNA repair genes in Wnt-addicted cells is minimally regulated by *FOXM1*. HPAF-II cells were transfected with two independent siRNAs against *FOXM1* or treated with ETC-159 (100 nM) for 48 h. Total RNA was isolated and expression of *FOXM1* and DNA repair genes was measured by qRT–PCR. The horizontal lines represent mean of replicates.

C Wnt-regulated HR and FA pathway gene expression is MYC-independent. Mice bearing HPAF-II xenografts without or with stabilized MYC (T58A) were treated with ETC-159 for 56 h. Tumors from the control and treated groups were harvested and the expression of DNA repair genes was measured.

D ChIP-seq data from K562 cells from ENCODE shows the binding of MYBL2 on the promoters of DNA repair genes but not on AXIN2. The putative MYBL2 motif locations identified using FIMO analysis are indicated (ENCODE Project Consortium, 2012).

E Enrichment of MYBL2 binding on the promoters of DNA repair genes. Chromatin immunoprecipitated from HPAF-II cells with IgG or MYBL2 antibody was analyzed using primers specific for the DNA binding sites in the indicated DNA repair genes. Representative images of the PCR products resolved on a 12% acrylamide gel are shown. The arrows indicate specific bands.

© 2021 The Authors

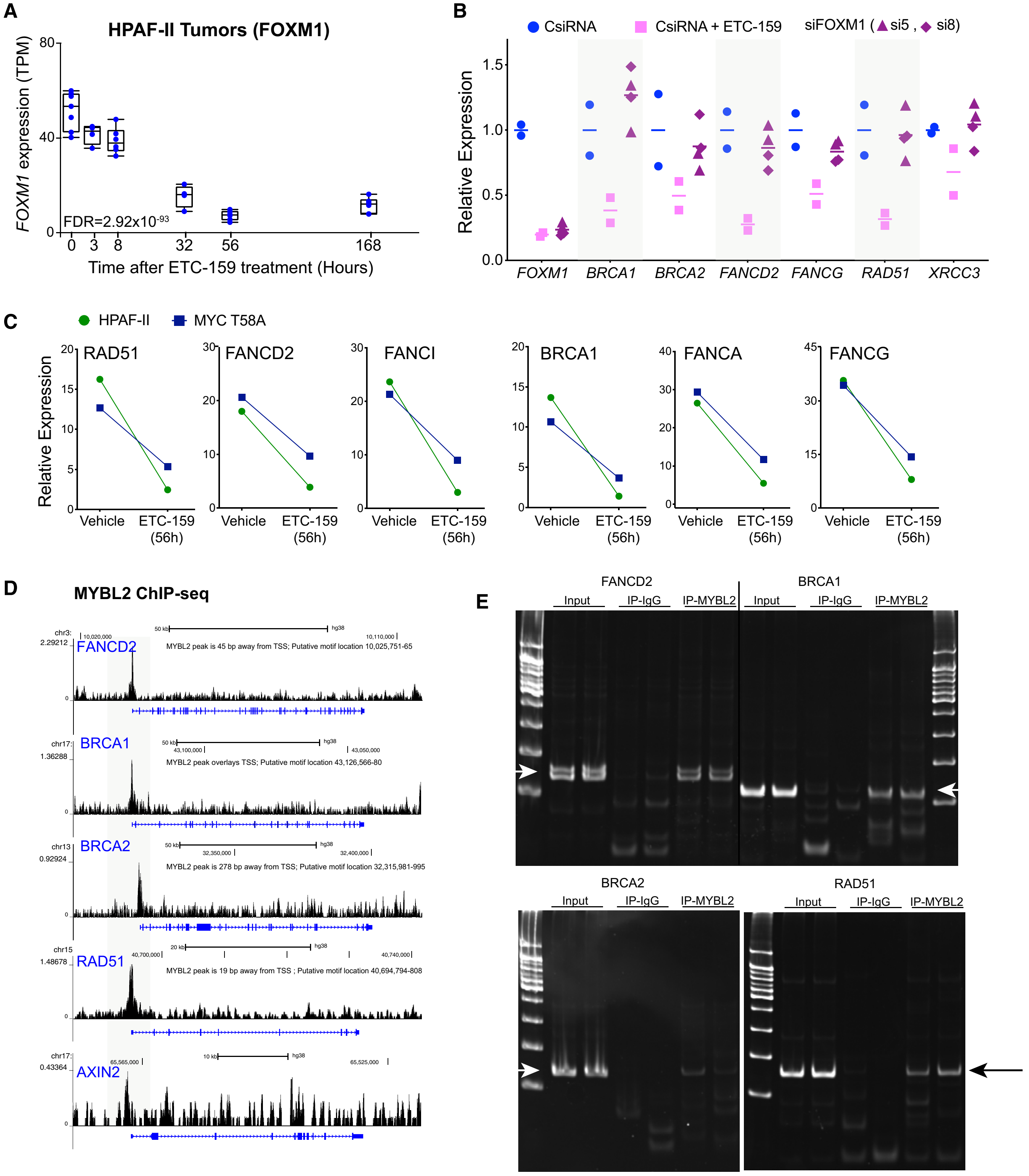
A
HPAF-II Tumors (FOXM1)
FOXM1 expression (TPM)
FDR=2.92x10-93
Time after ETC-159 treatment (Hours)
B
CsiRNA
CsiRNA + ETC-159
siFOXM1 (si5 , si8)
Relative Expression
FOXM1
BRCA1
BRCA2
FANCD2
FANCG
RAD51
XRCC3
C
HPAF-II
MYC T58A
Relative Expression
RAD51
FANCD2
FANCI
BRCA1
FANCA
FANCG
Vehicle
ETC-159 (56h)
D
MYBL2 ChIP-seq
FANCD2
MYBL2 peak is 45 bp away from TSS; Putative motif location 10,025,751-65
BRCA1
MYBL2 peak overlays TSS; Putative motif location 43,126,566-80
BRCA2
MYBL2 peak is 278 bp away from TSS; Putative motif location 32,315,981-995
RAD51
MYBL2 peak is 19 bp away from TSS ; Putative motif location 40,694,794-808
AXIN2
E
FANCD2
BRCA1
BRCA2
RAD51
Input
IP-IgG
IP-MYBL2

Figure EV2.

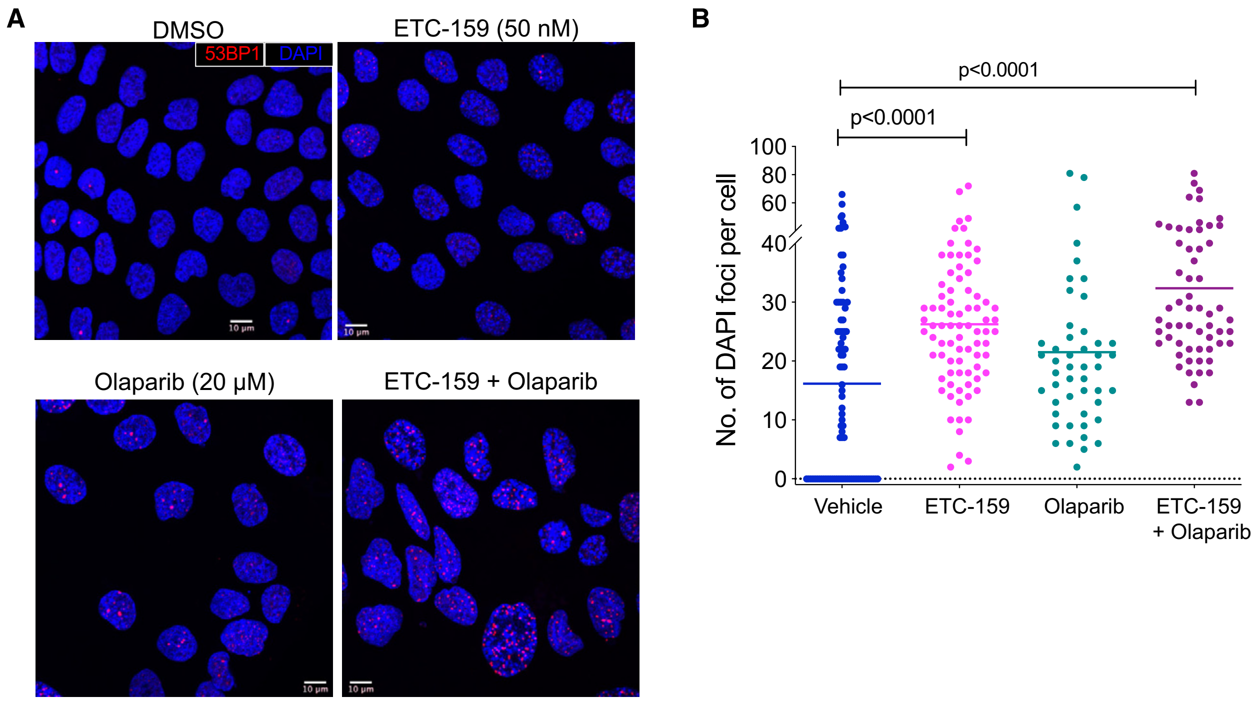

Figure EV3. (accompanying Fig 6).

A Representative images showing 53BP1 foci and senescence associated heterochromatin foci in HPAF-II treated with DMSO, ETC-159 (50 nM), olaparib (20 μM), or both for 7 days. 53BP1 foci (red) and nuclei counterstained with DAPI (blue).

B Wnt inhibition induces senescence associated heterochromatin foci, that is further enhanced by co-treatment with olaparib. HPAF-II cells were treated with DMSO, ETC-159 (50 nM), olaparib (20 μM) or both for 7 days and the number of SAHF per cell was assessed by DAPI fluorescent staining. The horizontal lines represent mean of replicates. *P*-values were calculated by Mann–Whitney *U*-test.

Figure EV4. (accompanying Fig 7).

HR and FA pathway genes are expressed in the Wnt high compartment of the small intestine: Immunohistochemical staining of sections of small intestine from C57BL/6J mice shows that the expression of *Brca1*, *Fancd2*, and *Rad51* is high in the crypts (Wnt high compartment) but not the villi.

 © 2021 The Authors

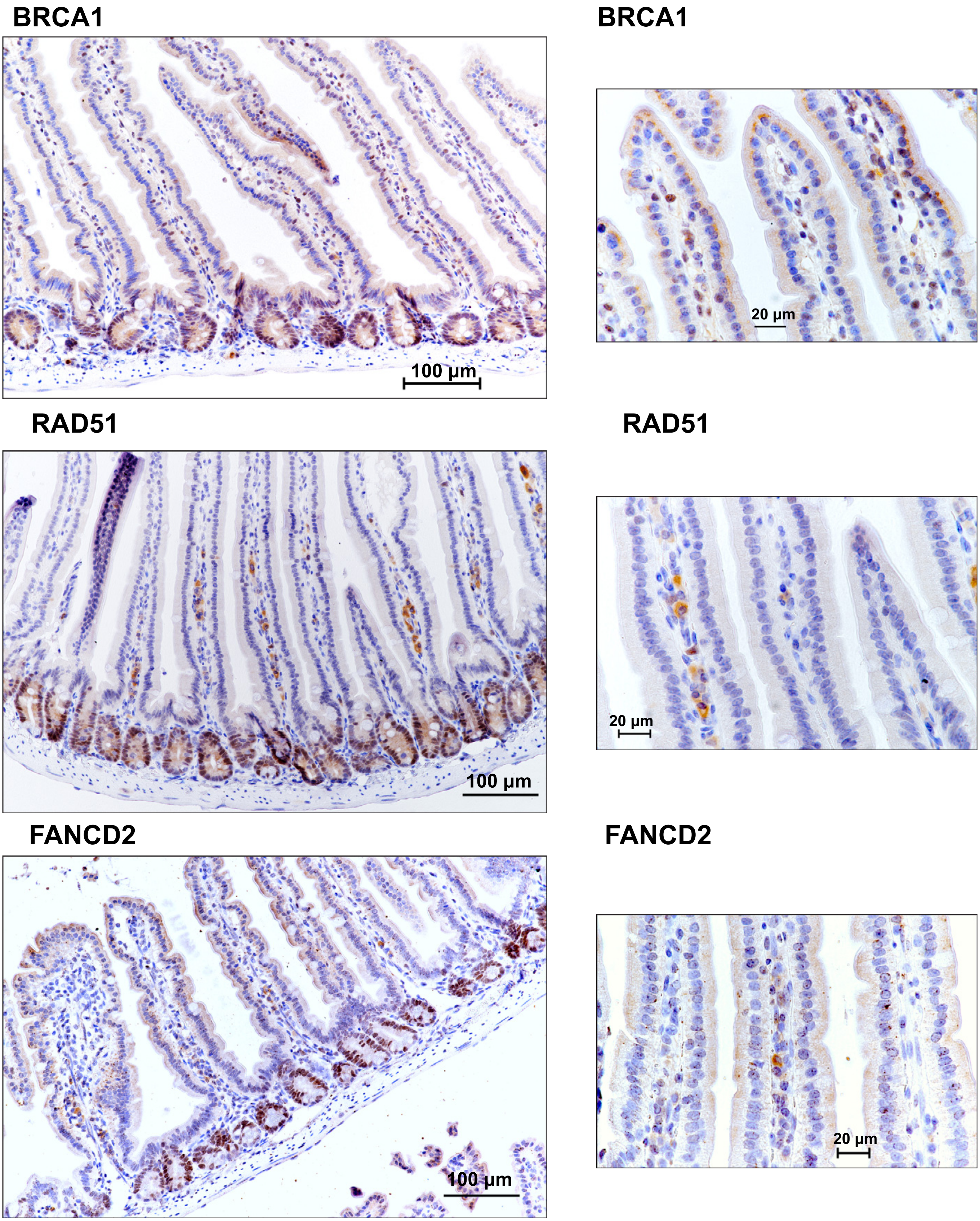
BRCA1
100 µm
BRCA1
20 µm
RAD51
100 µm
RAD51
20 µm
FANCD2
100 µm
FANCD2
20 µm

Figure EV4.

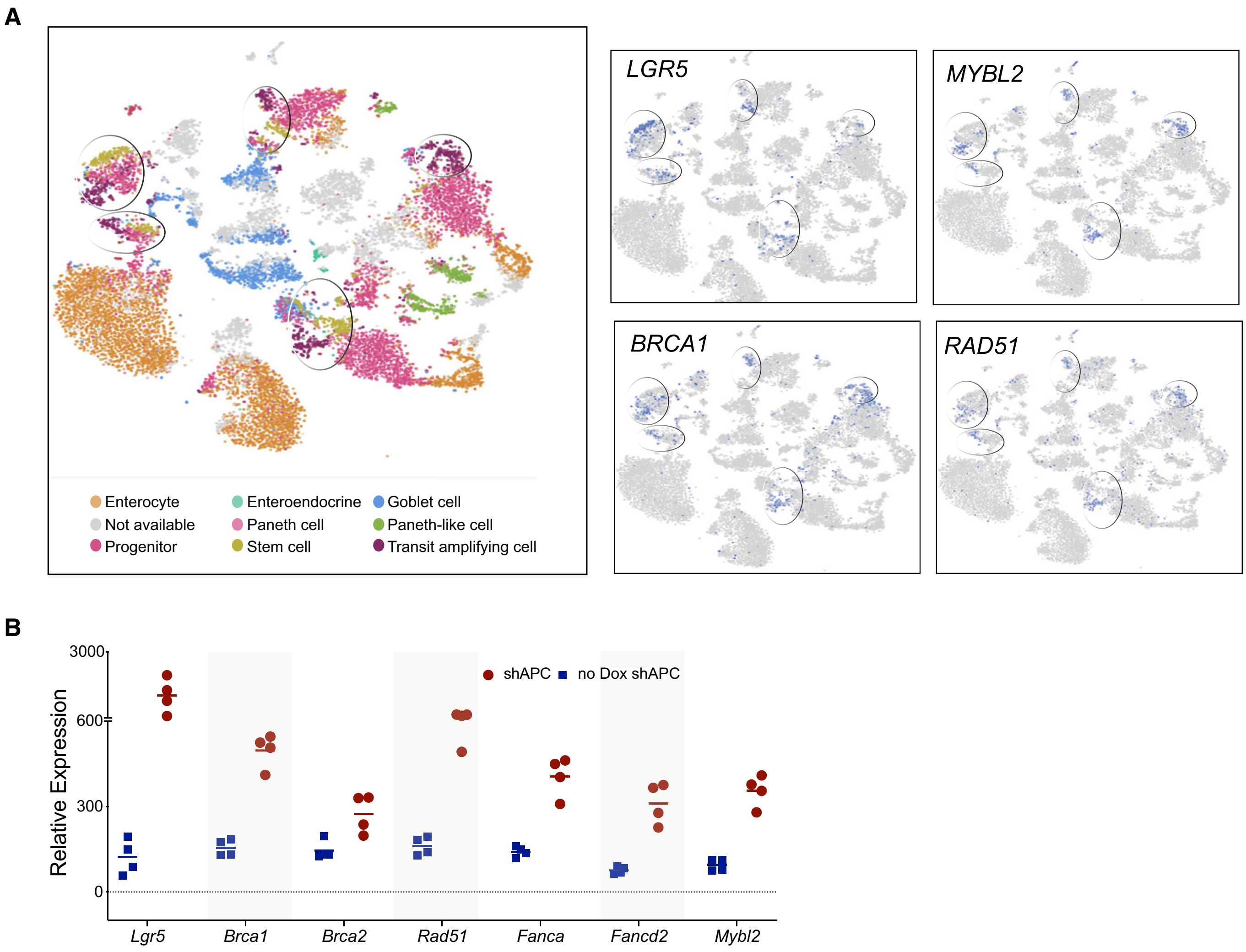

Figure EV5. (accompanying Fig 7).

A Expression of MYBL2 and DNA repair genes is high in the stem and transit-amplifying cells. Left: Analysis of single-cell RNA-seq data from human intestine identifies multiple cell type-specific clusters as indicated with stem and transit-amplifying clusters circled (Wang *et al*, 2020). Right: *LGR5*, *MYBL2*, *BRCA*1, and *RAD51* genes are high in these clusters. From https://www.ebi.ac.uk/gxa/sc/experiments/E-GEOD-125970/results/.

B Knockdown of APC leads to an increase in Mybl2, Brca, and FA gene expression. Gene expression analysis in intestinal organoids where dox-inducible APC knockdown produced high Wnt signaling. Organoids treated with and without doxycycline were compared (Dow *et al*, 2015). Increased Wnt signaling due to shAPC increased the expression of *Lgr5* as well as *Mybl2*, *Brca1/2*, and other HR/FA genes. The horizontal lines represent mean of replicates.

 © 2021 The Authors
