# Supplementary material for: WNT inhibition creates a BRCA‐like state in Wnt‐addicted cancer
Source: EMBO Mol Med. 2021 Mar 4;13(4):e13349. doi: 10.15252/emmm.202013349 (PMC8033517; doi:10.15252/emmm.202013349)
Supplement: Supplementary file 2 — Table EV1 [file EMMM-13-e13349-s004.pdf]

Table EV1: IC_{50} of the indicated drugs for the respective cell lines in soft agar or low density plating assays.

Cell Line	**ETC-159 ED_{50}**	**Olaparib ED_{50}**
HPAF-II	0.0077 µM	39.19 µM
EGI-1	0.0275 µM	8.65 µM
MCAS	0.287 µM	3.979 µM
CFPAC-1	0.027 µM	6.25 µM
PaTu8988T	1.1 µM	1.01 µM
COLO 320HSR	0.5 µM (G007-LK)	2.0 µM
