# Supplementary material for: WNT inhibition creates a BRCA‐like state in Wnt‐addicted cancer
Source: EMBO Mol Med. 2021 Mar 4;13(4):e13349. doi: 10.15252/emmm.202013349 (PMC8033517; doi:10.15252/emmm.202013349)
Supplement: Supplementary file 3 — Table EV2 [file EMMM-13-e13349-s003.pdf]

Table EV2: List of qRT-PCR primers

Gene	Species	Forward primer (5'-3')	Reverse primer (5'-3')
Axin2	Human, Mouse	CTCCCCACCTTGAATGAAGA	TGGCTGGTGCAAAGACATAG
BRCA1	Human	CAACATGCCCACAGATCAAC	ATGGAAGCCATTGTCCTCTG
BRCA2	Human	CAGAAGCCCTTTGAGAGTGG	TCCATCTGGGCTCCATTTAG
FANCD2	Human	CCCTGAGCTGCTTTTCTTGC	CGGCTTCCTTTGTTCTTGAG
FANCG	Human	CTGTTCTTCCCTTGGAGCTG	TCTCTAGGCTCCGCTGGATA
RAD51	Human	TTTGGAGAATTCCGAACTGG	TACATGGCCTTTCCTTCAC
XRCC3	Human	GTGCATCAACCAGGTGACAG	TTAGCCCAGGTTATGCCAAG
CTNNB1	Human	ATGGCTTGGAATGAGACTGCT	CCCATCAACTGGATAGTCAGC
MYBL2	Human	GAATTCCCGAAGCGTGAGGA	CAGGGTCCGACTCGATCAAG
FOXM1	Human	GAGCAGCGACAGGTTAAGGT	GTCATGCGCTTCCTCTCAGT
CDKN2B	Human	GGAAAGAAGGGAAGAGTGTCGTT	CGCGCATTCCGCAGC
LMNB1	Human	GATTGCCCAGTTGGAAGCCT	TGGTCTCGTTAATCTCCTCTTCATACA
IL32	Human	TGGCGGCTTATTATGAGGAGC	CTCGGCACCGTAATCCATCTC
CCN2	Human	TTGGCCCAGACCCAACTATG	CAGGAGGCGTTGTCATTGGT
MKI67	Human	AGTGCGAAGGTTCTCATGCA	TGCACACCTCTTGACACTCC
AURKB	Human	GAAGGTGGATCTGTGGTGCA	CGGGGAACTTTAGGTCCACC
CDK1	Human	TCCCAATAATGAAGTGTGGCCA	TGCCATTTTGCCAGAAATTCGT
CCNE2	Human	GCCGTTTACAAGCTAAGCAG	ACAGGTGGCCAACAATTCCT
ACTB	Human	ATAGCACAGCCTGGATAGCAACGTAC	CACCTTCTACAATGAGCTGCGTGTG
EPN1	Human	CTCTGACTTTGACCGACTCC	TGACCCCACTCATGTCAAAC
Brca1	Mouse	CCGGATACGAGAGTGAAACAA	TGCTGCAGCTTTATCAGGTT
Brca2	Mouse	AGGAAATGTTGGCTGTGTGGA	CGCTGTGTTGTGTCTTCTTCG
Fancd2	Mouse	CAAAATCAGCTAGGTGTGGATCA	CCAGGCCATTAACAAACTCTTCT
Fanca	Mouse	GTGGTCGGTGGATGAGATGTT	CCTAACTCCTCTCCACGCAAA
Rad51	Mouse	AAGTTTTGGTCCACAGCCTATTT	CGGTGCATAAGCAACAGCC
Mybl2	Mouse	GTGAGGCAGTTTGGACAGCAA	GGATTCAAAACCCTCAGCCA
Pgk1	Mouse	TCAAAAGCGCACGTCTGCCG	AAGTCCACCCTCATCACGACCC
Epn1	Mouse	TTGTGAGTCGCGTCATTTCTC	CCTCTGAGTAGTTGTGGACGATA
DRGFP-CN1	-	AGATCCGCCACAACATCGAG	TCTCGTTGGGGTCTTTGCTC
DRGFP-CN2	-	AGCAAAGACCCCAACGAGAA	TCGTCCATGCCGAGAGTGAT
ZNF80	Human	CTGTGACCTGCAGCTCATCCT	TAAGTTCTCTGACGTTGACTGATGTG
GPR15	Human	GGTCCCTGGTGGCCTTAATT	TTGCTGGTAATGGGCACACA
BRCA1 ChIP	Human	CCCATCTGTCAGCTTCGGAA	CCTCTGCTCTGGGTAAAGGT
BRCA2 ChIP	Human	TTGTAAGATCGGCTCGCTTT	TTTGCTCCAGCTCATGTTTG
FANCD2 ChIP	Human	CGGCTTCTCGGTGAGTAAGT	TCTCAGGGCAGATGAGGAAG
RAD51 ChIP	Human	CGAACTCCTAGGCTCAGACG	TTCCCTTTTTGCGTAAGGAC
